# Phase Transformation on Two-Dimensional MoTe_2_ Films for Surface-Enhanced Raman Spectroscopy

**DOI:** 10.3390/molecules29215216

**Published:** 2024-11-04

**Authors:** Caiye Zhao, Junwen Huang

**Affiliations:** School of Mechanics and Optoelectronic Physics, Anhui University of Science and Technology, Huainan 232001, China; t58001115@gmail.com

**Keywords:** phase transformation, molybdenum telluride, surface-enhanced Raman spectroscopy, charge transfer

## Abstract

Two-dimensional (2D) transition metal dichalcogenides (TMDs) have recently become attractive candidate substrates for surface-enhanced Raman spectroscopy (SERS) owing to their atomically flat surfaces and adjustable electronic properties. Herein, large-scale 2D 1T′- and 2H-MoTe_2_ films were prepared using a chemical vapor deposition method. We found that phase structure plays an important role in the enhancement of the SERS performances of MoTe_2_ films. 1T′-MoTe_2_ films showed a strong SERS effect with a detection limit of 1 × 10^−9^ M for the R6G molecule, which is one order of magnitude lower than that of 2H-MoTe_2_ films. We demonstrated that the SERS sensitivity of MoTe_2_ films is derived from the efficient photoinduced charge transfer process between MoTe_2_ and adsorbed molecules. Moreover, a prohibited fish drug could be detected by using 1T′-MoTe_2_ films as SERS substrates. Our study paves the way to the development and application of high-performance SERS substrates based on TMD phase engineering.

## 1. Introduction

The origins of surface-enhanced Raman spectroscopy (SERS) can be traced back to 1974 when Fleischmann et al. discovered the enhanced Raman signals of pyridine molecules on an electrochemically roughened silver electrode [1]. As a powerful trace analysis technique with highly sensitive, label-free, and non-destructive detection, SERS has been successfully applied in bioanalysis, catalysis, food safety, environmental monitoring, and other fields [2,3,4,5,6]. Currently, there are two main mechanisms used to enhance SERS: the electromagnetic enhancement mechanism (EM) and the chemical enhancement mechanism (CM). Conventionally, noble metals (gold or silver) with roughened surface morphologies are widely used SERS materials, which exhibit high SERS sensitivity owing to the EM caused by a strong localized surface plasmon resonance (LSPR) effect [7]. However, their high price, poor signal uniformity, and complicated “hotspots” (e.g., high-intensity electromagnetic field regions formed at nanoscale gaps) regulation limit their widespread applications [6]. Hence, the development of high-performance noble metal-free SERS substrates is urgently needed.

In the last ten years, noble metal-free SERS materials, such as graphene [8], transition metal oxides [9,10], transition metal carbides or nitrides (MXenes) [11], transition metal borides (MBenes) [12], metal–organic frameworks [13], perovskites [14], graphdiyne [15], and organic semiconductors [16], have received great attention. The SERS effect in these noble metal-free materials is mainly ascribed to the CM, which originates from the charge transfer (CT) between the materials and the adsorbed molecules [2]. In particular, two-dimensional (2D) transition metal dichalcogenide (TMD) materials with layered features are considered as promising SERS materials to replace the noble metals due to their atomically flat surfaces, superior stability, biocompatibility, adjustable electronic properties, and ease of fabrication [6]. Until now, various TMD materials such as MoS_2_ [17], MoSe_2_ [18], MoTe_2_ [19], WS_2_ [20], WSe_2_ [21], TaSe_2_ [22], and NbS_2_ [23] have been used as SERS materials. For instance, Meng et al. developed WS_2_ SERS substrates with a limit of detection (LOD) as low as 10^−7^ M for rhodamine 6G (R6G) molecules [24]. Ge et al. reported that atomically thin TaSe_2_ films exhibited excellent SERS performance with an LOD of 1 × 10^−10^ M for R6G molecules [22]. In order to improve the SERS sensitivity of TMD materials, some strategies such as defect engineering [21], alloy engineering [25], phase engineering [20], and heterojunction engineering [26] have been developed. For example, Tang et al. reported an alloy engineering strategy to improve the SERS efficiency of a target molecule by manipulating the energy levels of the SERS substrates [25]. Guan et al. reported phase-engineered 2M-WS_2_ SERS substrates exhibited an LOD for crystal violet (CV) molecules of 10^−8^ M by tuning the Fermi level of WS_2_ [20]. Lan et al. designed WN/monolayer MoS_2_ heterostructure chips with an LOD as low as 10^−10^ M for R6G molecules [27]. However, the effect of phase transformation on chemical vapor deposition (CVD)-grown large-area 2D MoTe_2_-based SERS substrates is rarely reported.

Herein, we synthesized large-scale 2D MoTe_2_ films with different phases (1T′ and 2H phases) using a CVD method. We found that 1T′-MoTe_2_ films have higher SERS sensitivity than 2H-MoTe_2_ films. The Raman enhancement factor (EF) of 1T′-MoTe_2_ films’ detection of R6G molecules could be up to 1.9 × 10^6^ with a limit of detection up to 10^−9^ M, which could be comparable to noble metal SERS substrates. The underlying enhancement mechanisms of these MoTe_2_ films were also explained. In addition, a prohibited fish drug was detectable on 1T′-MoTe_2_ films, indicating the potential of 1T′-MoTe_2_ films in addressing the problems of food safety. This work provides the way to design high-performance TMD-based SERS substrates.

## 2. Results and Discussion

### 2.1. Characterization of MoTe_2_ Films

**Figure 1:**
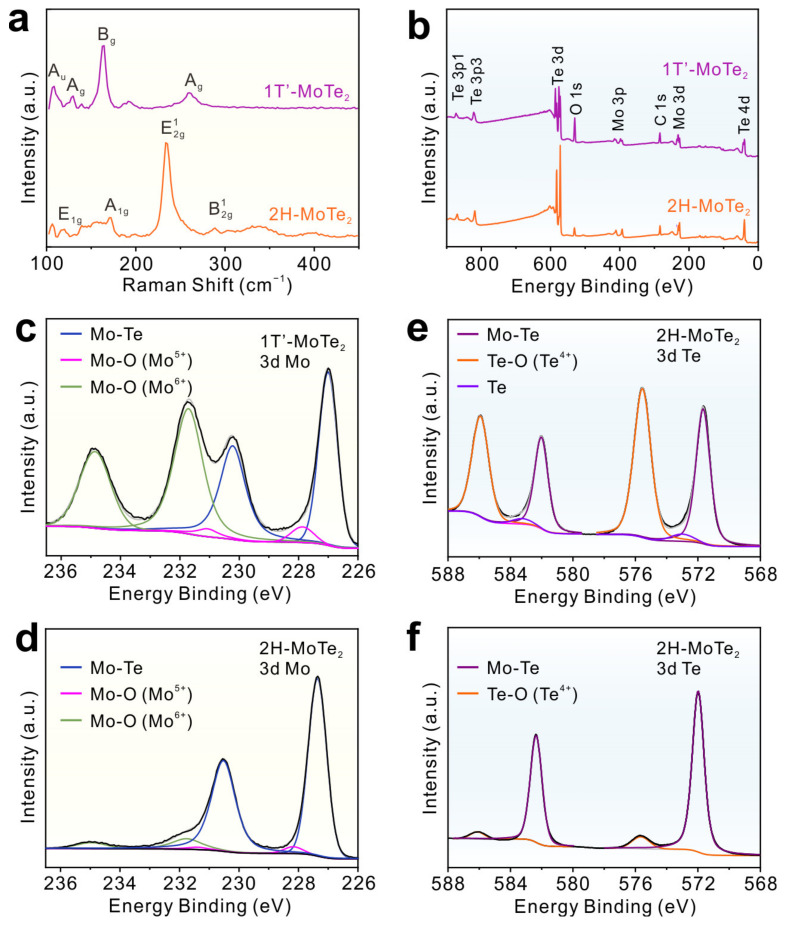
(**a**) Raman spectra of 1T′- and 2H-MoTe_2_ films. (**b**) The survey spectra of 1T′- and 2H-MoTe_2_ films. (**c**,**d**) The Mo 3d XPS spectra of 1T′- and 2H-MoTe_2_ films. (**e**,**f**) The Te 3d XPS spectra of 1T′- and 2H-MoTe_2_ films.

**Figure 2:**
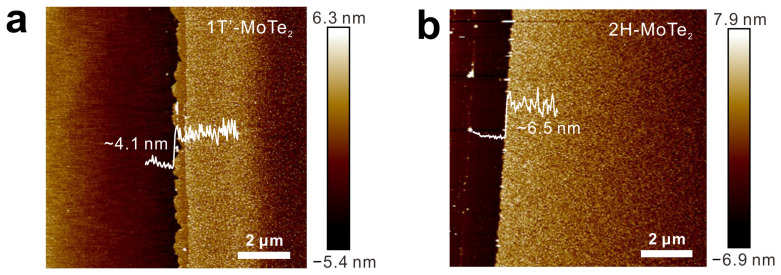
(**a**,**b**) AFM images of (**a**) 1T′- and (**b**) 2H-MoTe_2_ films. The white lines are the height profiles.

The structures of MoTe_2_ films were characterized by using Raman spectroscopy first. In the Raman spectra of 1T′-MoTe_2_ films (Figure 1a), five characteristic Raman peaks of 1T′-MoTe_2_ at 107.7 (A_u_), 129.7 (A_g_), 162.5 (B_g_), 191.4 (B_g_), and 260.2 cm^−1^ (A_g_) were observed [28,29]. For 2H-MoTe_2_ films, the four Raman peaks at 118.1, 171.3, 234.5, and 288.8 cm^−1^ originated from the E_1g_, A_1g_, E^1^_2g_, and B^1^_2g_ vibration modes of 2H-MoTe_2_ [29]. XPS was used to analyze the elemental compositions and chemical states of MoTe_2_ films. In the survey XPS spectra of MoTe_2_ films, the signals of Mo, Te, and O elements were observed (Figure 1b). For 1T′-MoTe_2_, the high-resolution Mo 3d core-level photoemission peak could be fitted with three doublet peaks, which were attributed to Mo-Te (227.0, 230.2 eV), Mo-O (Mo^5+^) (227.8, 231.0 eV), and Mo-O (Mo^6+^) (231.7, 234.9 eV), respectively (Figure 1c) [30]. As shown in Figure 1d, the Mo 3d XPS spectra of 2H-MoTe_2_ could be fitted by three spin–orbit doublets, corresponding to Mo-Te (227.3, 230.5 eV), Mo-O (Mo^5+^) (228.1, 231.3 eV), and Mo-O (Mo^6+^) (231.8, 235.0 eV), respectively. Compared with 1T′-MoTe_2_ films, there was a shift (~0.3 eV) to a higher binding energy for 2H-MoTe_2_ films, which is consistent with previous work [29]. In Figure 1e, the Te 3d XPS spectra of 1T′-MoTe_2_ exhibit three spin–orbit doublets, corresponding to Mo-Te (571.7, 582.0 eV), Te (572.9, 583.2 eV), and Te-O (575.6, 586.0 eV), respectively [31]. The two spin–orbit doublets of Mo-Te (572.0, 582.3 eV) and Te-O (575.7, 586.1 eV) were observed in 2H-MoTe_2_ films (Figure 1f). According to the XPS results, the rate of oxidized Mo on the surface of 1T’- and 2H-MoTe_2_ was 54.2% and 12.0%, receptively. The rate of oxidized Te on the surface of 1T’- and 2H-MoTe_2_ was 56.7% and 7.7%, respectively. The higher oxidation of 1T′-MoTe_2_ can be attributed to the metastable 1T′ phase [32]. The MoTe_2_ films were further characterized using an AFM. The AFM characterizations showed that the thicknesses of the 1T′- and 2H-MoTe_2_ films were about 4.1 and 6.5 nm, respectively (Figure 2a,b). All the results indicate that the 2D 1T′- and 2H-MoTe_2_ films were synthesized successfully.

### 2.2. SERS Performances of MoTe_2_ Films

**Figure 3:**
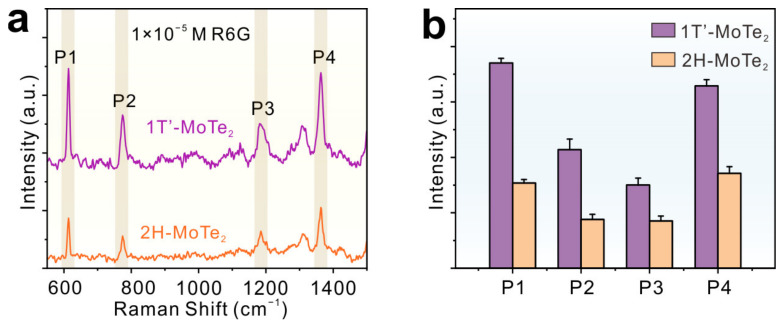
(**a**,**b**) The Raman spectra and corresponding signal intensity of 1 × 10^−5^ M R6G adsorbed on 1T′- and 2H-MoTe_2_ films.

In order to characterize the Raman enhancement effect on MoTe_2_ films, R6G was chosen as the probe molecule and the excitation wavelength was 532 nm. In Figure 3a, the main Raman characteristic peaks for R6G (1 × 10^−5^ M) molecules adsorbed on MoTe_2_ can be observed at 612 (P1, C-C-C ring in-plane bend), 773 (P2, C-H out-of-plane bend), 1185 (P3, C-C stretching vibration bend), and 1363 cm^−1^ (P4, aromatic C-C stretching), respectively [33]. By monitoring the Raman peak at 612 cm^−1^, the Raman intensity of R6G molecules on 1T′-MoTe_2_ was shown to be ~2.4 times stronger than on 2H-MoTe_2_ (Figure 3b). These results indicate that 1T′-MoTe_2_ films have higher SERS sensitivity than 2H-MoTe_2_ films.

**Figure 4:**
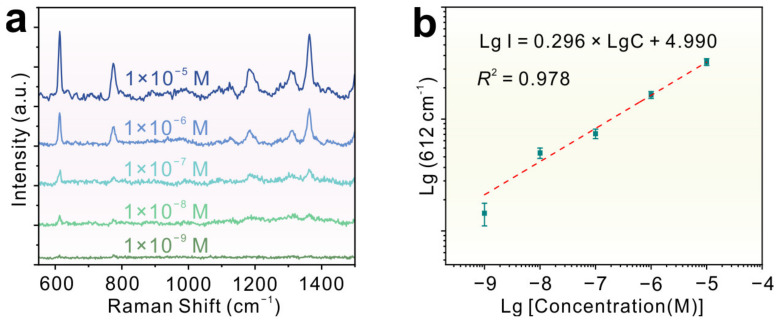
(**a**) The Raman spectra of R6G with different concentrations ranging from 1 × 10^−5^ to 1 × 10^−9^ M adsorbed on 1T′-MoTe_2_ films. (**b**) Logarithm of integral Raman intensity at 612 cm^−1^ as a function of molecular concentration, and corresponding fitting curves (the red dotted line).

Furthermore, a series of different concentrations of R6G ranging from 1 × 10^−5^ to 1 × 10^−9^ M was adsorbed on 1T′-MoTe_2_ (Figure 4a). The Raman signals of R6G can still be detectable even down to the concentration of 1 × 10^−9^ M, which is one order of magnitude lower compared with 2H-MoTe_2_ (Figure S1). Figure 4b shows the working curves of the R6G concentration versus the Raman intensity in their logarithm scale. A well-defined linear response to the molecular concentrations in the range of 1 × 10^−5^ to 1 × 10^−9^ M with a correlation coefficient (*R*^2^) of 0.978 was obtained, which indicated 1T′-MoTe_2_ films have great potential for SERS quantitative analysis. The Raman EF could be calculated by using the follow formula [34]:

(1) $$\mathrm{EF}=\frac{I_{\mathrm{SERS}}}{I_{\mathrm{Raman}}}\times \frac{N_{\mathrm{Raman}}}{N_{\mathrm{SERS}}},$$

in which *I*_Raman_ and *I*_SERS_ are the intensities of the selected Raman peak in the normal Raman spectra and SERS, respectively. *N*_Raman_ and *N*_SERS_ are the average number of molecules in the scattering area for normal Raman and SERS measurements, respectively. The corresponding EFs of the 2H- and 1T′-MoTe_2_ films for R6G were calculated to be 5.2 × 10^5^ and 1.9 × 10^6^, respectively (details of the calculation process can be found in the Supplementary Materials and Figure S2). The SERS sensitivity of 1T′-MoTe_2_ films could be comparable to those of reported 2D materials (Table S1) [10,20,22,24,25,33,34,35,36,37,38,39].

**Figure 5:**
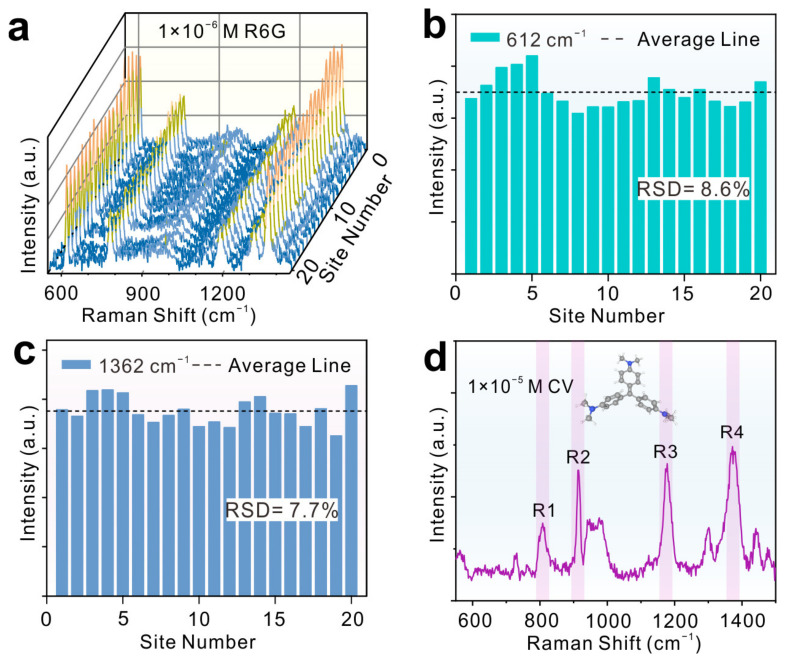
(**a**) The Raman spectra of R6G (1 × 10^−6^ M) collected from 20 random sites. High and low Raman intensities are shown in yellow and blue, respectively. (**b**,**c**) Raman intensity columns of peaks at 612 and 1362 cm^−1^ derived from (**a**). (**d**) The Raman spectra of 1 × 10^−5^ M CV adsorbed on 1T′-MoTe_2_ films.

The relative standard deviations (RSDs) were calculated by randomly selecting 20 points on 1T′-MoTe_2_ films (Figure 5a). The RSD values of characteristic Raman peaks at 612 and 1360 cm^−1^ were 8.6% and 7.7% (Figure 5b,c), respectively, indicating the good signal uniformity. In addition, CV is a prohibited fish drug, which can be detected by using 1T′-MoTe_2_ as a SERS substrate. As shown in Figure 5d, four characteristic Raman peaks of CV at 808 (R1, ring C-H bend), 915 (R2, ring skeletal vibration of radical orientation), 1178 (R3, ring C-H bend), and 1372 cm^−1^ (R4, N–phenyl stretching) could be observed [40], indicating that MoTe_2_ has a promising application in food safety.

### 2.3. Raman Enhancement Mechanism of MoTe_2_ Films

**Figure 6:**
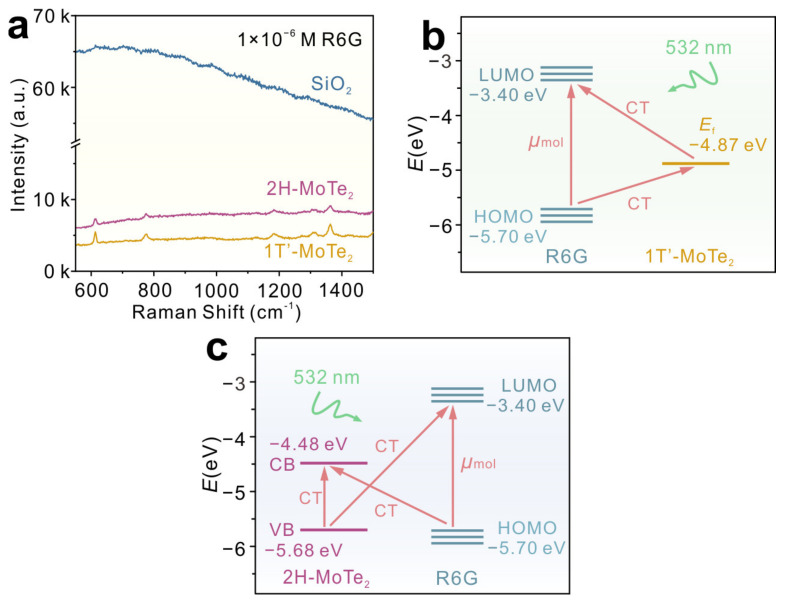
(**a**) Raman spectra of R6G (1 × 10^−6^ M) adsorbed on bare SiO_2_, 1T′-, and 2H-MoTe_2_ films without baseline correction. (**b**,**c**) The schematic energy level diagrams and CT processes in the (**b**) 1T′-MoTe_2_-R6G and (**c**) 2H-MoTe_2_-R6G systems. The arrows are the paths of CT processes.

The electromagnetic enhancement contribution is excluded in the MoTe_2_-induced SERS effect, because the LSPR of MoTe_2_ can hardly be excited at this laser wavelength (532 nm) [19]. Figure 6a shows the Raman spectra of R6G adsorbed on bare SiO_2_, 1T′-, and 2H-MoTe_2_ films without baseline correction. A large fluorescence background was observed on bare SiO_2_, and the Raman signals of R6G could not be detected. However, the fluorescence background was suppressed when the R6G molecules adsorbed on MoTe_2_ films. The strong quenching effects provide the evidence for the efficient CT processes between R6G and MoTe_2_ [15]. In addition, no Raman signal of the R6G molecule was observed on pristine Mo and Te films (Figure S3). The Raman intensities of molecules are proportional to the square of the polarizability tensor α, which can be expressed as α = A + B + C, where the A-term can only amplify the totally symmetric Raman modes, which follows the Franck–Condon selection rules. The B-term and C-term represent the CT from the molecules to the substrates and the CT process from the substrates to the molecules, respectively [2]. The schematic energy level diagrams and CT processes in the 1T-MoTe_2_-R6G system under the irradiation of a 532 nm laser are shown in Figure 6b,c. The highest occupied molecular orbital (HOMO) and the lowest unoccupied molecular orbital (LUMO) levels of R6G are at −3.40 and −5.70 eV, respectively. The Fermi level of 1T′-MoTe_2_ is located at −4.87 eV [41]. The conduction band (CB) and valence band (VB) of 2H-MoTe_2_ are at −4.48 eV and −5.68 eV, respectively [42]. In the 1T′-MoTe_2_-R6G system, the excited electrons can be transferred from the Fermi level of 1T′-MoTe_2_ to the HOMO of R6G, but also from the LOMO level of R6G to the Fermi level of 1T′-MoTe_2_ (Figure 6b). In the 2H-MoTe_2_-R6G system, the transfer of excited electrons from the HOMO level of R6G to the CB of 2H-MoTe_2_, from the VB of 2H-MoTe_2_ to the LOMO level of R6G, and from the CB of 2H-MoTe_2_ to the VB of 2H-MoTe_2_ could occur (Figure 6c). According to Herzberg–Teller coupling theory, A-term in the sum of the B-term or C-term can be described as [6]

(2) $$R_{\mathrm{mol}-\mathrm{CT}}\left(\omega \right)=\frac{\mu_{\mathrm{mol}}\mu_{\mathrm{CT}}h_{\mathrm{mol}-\mathrm{CT}}\langle i|Q_{K}|f\rangle }{\left({\left(\varepsilon_{1}\left(\omega \right)+2\varepsilon_{0}\right)}^{2}+\varepsilon_{2}^{2}\right)\left(\left(\omega_{\mathrm{CT}}^{2}-\omega^{2}\right)+\gamma_{\mathrm{CT}}^{2}\right)\left(\left(\omega_{\mathrm{mol}}^{2}-\omega^{2}\right)+\gamma_{\mathrm{mol}}^{2}\right)},$$

where *ω* is the frequency of the incident photons and *ω*_mol_ represents the molecular resonance frequency. Since the photon energy of a 532 nm laser (2.33 eV) is approximately equal to the band gap of R6G (*ω* ≈ *ω*_mol_), the molecular transition (*μ*_mol_) can be excited, which can further increase the Raman scattering cross-section.

AFM characterizations showed that 1T′-MoTe_2_ films with different thicknesses were prepared (Figure 2a and Figure S4). The thickness-dependent Raman enhancement of 1T′-MoTe_2_ films is shown in Figure S5. Compared with 16.5 nm thick 1T′-MoTe_2_ films (Figure S4), the 4.1 nm thick 1T′-MoTe_2_ films exhibited a larger SERS enhancement. The enhanced Raman intensities of R6G at 612 cm^−1^ on 4.1 nm thick 1T′-MoTe_2_ are ~3.7 times higher than those on 16.5 nm thick 1T′-MoTe_2_ films. According to Fermi’s golden rule, an abundant density of electronic states (DOS) near the Fermi level will give rise to a high electron transition probability between the adsorbed molecules and the SERS substrates [6]. Usually, few layered TMDs have a higher DOS near the Fermi level than multilayer TMDs [20,43]. So, the thinner 1T-MoTe_2_ films showed higher SERS sensitivity.

## 3. Experimental Section

### 3.1. Synthesis of MoTe_2_ Films

**Figure 7:**
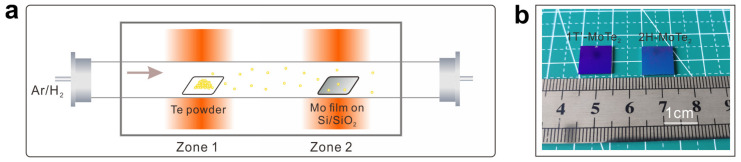
(**a**) Schematic illustration of the synthesis of MoTe_2_ films using the CVD method. (**b**) The optical images of as-prepared MoTe_2_ films.

MoTe_2_ films were prepared by tellurizing the Mo films (Figure 7a) [28]. A 2 nm Mo film was deposited on Si/SiO_2_ substrates by magnetron sputtering first. Then, the Mo films and high-purity Te powder (99.999%) were placed in one quartz tube. During the growth process, a mixture gas of Ar and H_2_ was flowed at rates of 4 and 5 sccm in a furnace. MoTe_2_ films with different structure phases were prepared by adjusting the reaction temperature and time. Typically, the growth temperature of 1T′-MoTe_2_ films was kept at 700 °C for 30 min. 1T′-MoTe_2_ films could be transformed into 2H-MoTe_2_ films by tellurizing the 1T′-MoTe_2_ films at 750 °C for 120 min. After that, the furnace was naturally cooled down to room temperature. The optical image of as-prepared MoTe_2_ films is shown in Figure 7b; large-scale (10 mm × 10 mm) MoTe_2_ films were prepared.

### 3.2. Characterization

The thicknesses of films were analyzed by using an atomic force microscope (AFM, Bruker Dimension Icon, Karlsruhe, Germany). UV–vis diffuse reflectance spectra were recorded using a UV–visible spectrophotometer (UV-2600, Shimadzu, Kyoto, Japan). X-ray photoelectron spectroscopy (XPS) spectra were recorded by a photoelectron spectrometer (ESCAlab250, Thermo Fisher Scientific, Waltham, MA, USA); the thickness explored with XPS in material is about 1 nm. Before Raman measurement, the MoTe_2_ films were immersed in the solutions of Raman probes with different concentrations (1 × 10^−5^~1 × 10^−9^ M) for 1 h and were dried by N_2_. All the Raman measurements were taken with a Renishaw microscopic confocal Raman spectrometer (InVia Qontor, London, UK). The excitation wavelength was 532 nm with a 600 gr mm^−1^ grating and a 50× objective was used to focus the laser beam. The integral times were all 50 s.

## 4. Conclusions

In summary, the phase structure-induced Raman enhancement of CVD-grown large-scale 2D MoTe_2_ films was demonstrated. A sensitive molecular sensing performance with a low LOD of 1 × 10^−9^ M for R6G molecules was achieved on the 2D 1T′-MoTe films, which was one order of magnitude lower than that of 2D 2H-MoTe_2_ films. We demonstrated that efficient charge transfer processes at the interface between the MoTe_2_ and the adsorbed molecules endow MoTe_2_ with high SERS sensitivity. Furthermore, MoTe_2_ also could be used to detect a prohibited fish drug. Our work’s results clearly demonstrate that the 2D MoTe_2_ films would be ideal candidates for next-generation SERS substrates.

## Data Availability

The original contributions presented in the study are included in the article; further inquiries can be directed to the corresponding author.

## Supplementary Materials

The following supporting information can be downloaded at https://www.mdpi.com/article/10.3390/molecules29215216/s1: Figure S1: The Raman spectra of 1 × 10^−8^ M R6G adsorbed on 2H-MoTe_2_ films; Figure S2: the Raman spectra of bulk R6G with 10 s acquisition time; Figure S3: the Raman spectra of 1 × 10^−5^ M R6G adsorbed on Mo and Te films; Figure S4: AFM image and thickness profiles of the thicker 1T′-MoTe_2_ films; Figure S5: the Raman spectra of 1 × 10^−5^ M R6G adsorbed on 1T′-MoTe_2_ films with different thicknesses; Table S1: SERS performances for various 2D materials have been reported.
