# Supplementary material for: Phase Transformation on Two-Dimensional MoTe2 Films for Surface-Enhanced Raman Spectroscopy
Source: Molecules. 2024 Nov 4;29(21):5216. doi: 10.3390/molecules29215216 (PMC11547442; doi:10.3390/molecules29215216)
Supplement: Supplementary file 1 [file molecules-29-05216-s001.zip › molecules-3270298-supplementary.pdf]

Supplementary Materials

Phase transformation on two-dimensional $MoTe_2$ films for surface-enhanced Raman spectroscopy

Caiye Zhao [*], Junwen Huang

School of Mechanics and Optoelectronic Physics, Anhui University of Science and Technology, Huainan 232001, China

[*] Authors to whom correspondence should be addressed: caiyezhao@163.com (C. Z.)

Calculation of the Raman enhancement factor (EF)

The Raman EF of the SERS substrates could be calculated using the follow equation [1,2]:

$$EF = (I_{SERS}/N_{SERS}) / (I_{Raman}/N_{Raman}) \quad (S1)$$

$$N_{SERS} = CVN_A A_{Raman}/A_{Sub} \quad (S2)$$

$$N_{Raman} = \rho h A_{Raman} N_A/M \quad (S3)$$

where N_{SERS} and N_{Raman} are the average number of molecules in scattering area for SERS and non-SERS measurement, respectively. I_{SERS} and I_{Raman} are the intensities of the selected Raman peak in the SERS and non-SERS spectra, respectively. The data of bulk R6G is used as non-SERS-active reference. C is the molar concentration of R6G solution and V is the volume of the droplet (10 μL). N_A is Avogadro constant (6.023×10^{23} mol^{-1}). A_{Raman} is the laser spot area (~0.79 μm^2). A_{Sub} is the effective area of the substrate, which is approximately 9 πmm^2. ρ is the density of bulk R6G (1.15 g cm^{-3}) and h is the confocal depth of the laser beam (21 μm). M is the molecular weight of R6G (479 g mol^{-1}).

Here, we use the R6G peak at 612 cm^{-1} to estimate the EF. The peak 612 cm^{-1} of bulk R6G is 1210 counts with 10 s acquisition time (Figure S2), and that of 1×10^{-9} M R6G is 84 counts with 50 s acquisition time. So, the Raman intensity ratio is estimated to be $I_{SERS}/I_{Raman} = (84/50) / (1210/10) = 1.39 \times 10^{-2}$ with normalization concerning the acquisition time. For the normal Raman measurement, The N_{Raman} value was 0.23×10^{11} by using the equation (S3). For the SERS measurement, the N_{SERS} value was 0.17×10^{3} by using the equation (S2). Finally, the Raman EFs of the 1T′-$MoTe_2$ is estimated to be ~1.9×10^{6} at the R6G concentration of 1×10^{-9} M by using the equation (S1).

Figures

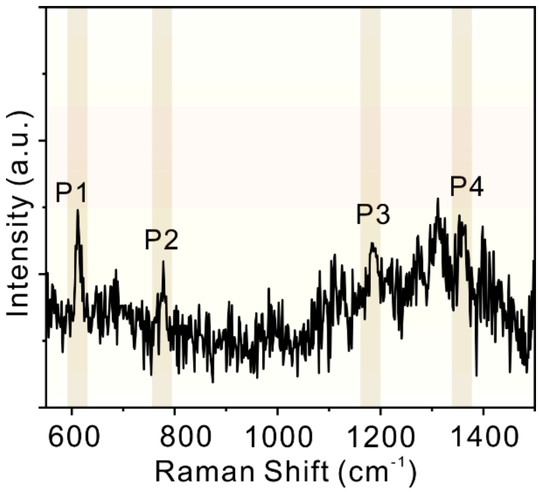

Figure S1. The Raman spectra of 1× 10^{-8} M R6G adsorbed on 2H-$MoTe_2$ films.

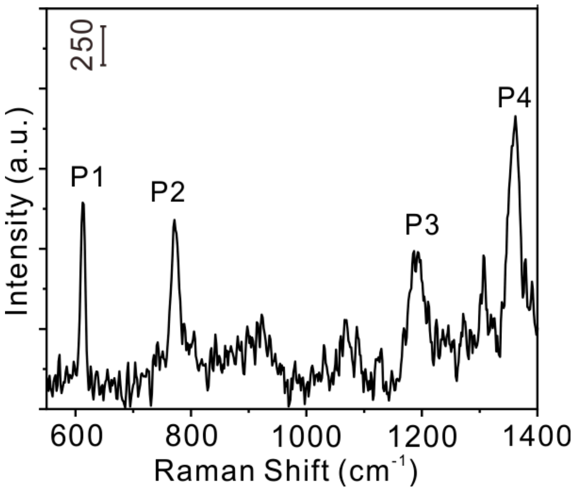

Figure S2. The Raman spectra of bulk R6G with 10 s acquisition time.

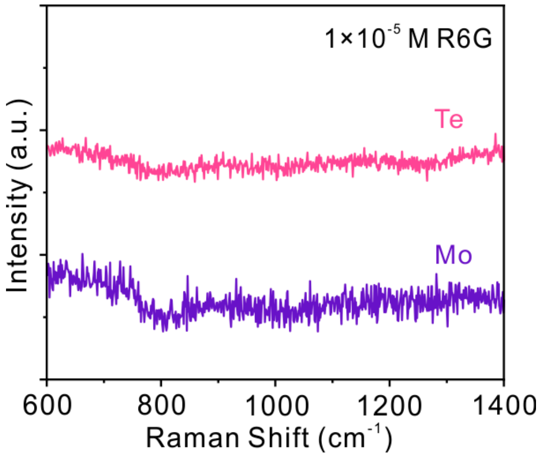

Figure S3. The Raman spectra of 1× 10^{-5} M R6G adsorbed on Mo and Te films.

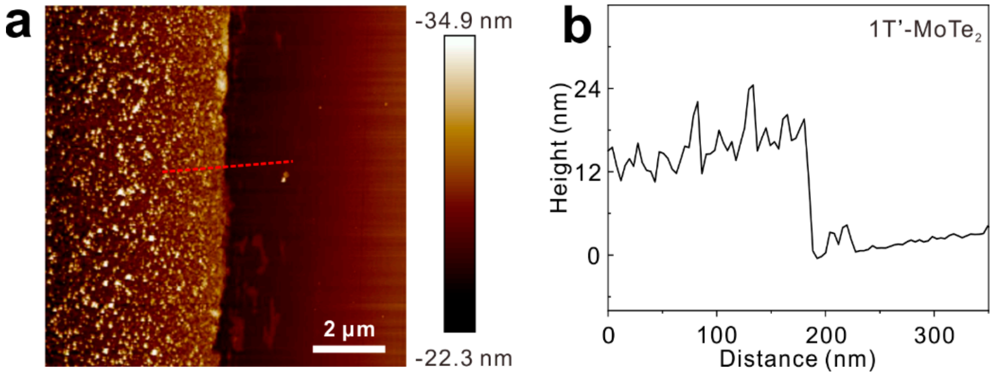

Figure S4. (**a**) AFM image of the thicker 1T′-$MoTe_2$ films. (b) Line profile across the dashed line in the image (a).

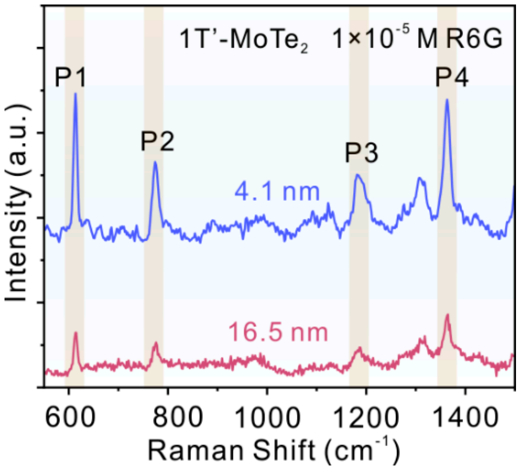

Figure S5. The Raman spectra of 1 × 10^{-5} M R6G adsorbed on 1T′-$MoTe_2$ films with different thicknesses.

Table S1. SERS performances for various 2D materials have been reported.

2D Substrate	Probe	LOD	EF	Author
2D BCN nanosheets	CV	1×10^{-7} M	N/A	Xe *et al.*[3]
2D $WS_{1.08}Se_{0.92}$	R6G	N/A	1.27×10^{6}	Tang *et al.*[4]
2D GaN flakes	CV	2×10^{-7} M	5.2×10^{5}	Zhao *et al.*[5]
$HfTe_2$ nanosheets	R6G	1×10^{-9} M	2.32×10^{6}	Liu *et al.*[6]
Graphene oxide	R6G	10^{-8} M	N/A	Li *et al.*[7]
2D MoO_{3-x} nanosheets	R6G	1×10^{-7} M	3.32×10^{5}	Lan *et al.*[8]
2D Mo_2C MXenes	R6G	1×10^{-7} M	1.20×10^{5}	Lan *et al.*[9]
2D $TaSe_2$ films	R6G	1×10^{-10} M	N/A	Ge *et al.*[10]
2D ReS_2 films	R6G	1×10^{-7} M	N/A	Wang *et al.*[11]
2D Ta_4C_3 MXenes	R6G	1×10^{-7} M	1.51×10^{5}	Lan *et al.*[12]
WS_2 films	R6G	1×10^{-7} M	N/A	Meng *et al.*[13]
Multilayer 2M-WS_2	CV	10^{-8} M	N/A	Zhao *et al.*[14]
2H-$MoTe_2$ films	R6G	1×10^{-8} M	5.2×10^{5}	**This work**
1T′-$MoTe_2$ films	R6G	1×10^{-9} M	1.9×10^{6}	**This work**

References

1. Cong, S.; Yuan, Y.; Chen, Z.; Hou, J.; Yang, M.; Su, Y.; Zhang, Y.; Li, L.; Li, Q.; Geng, F.; Zhao, Z. Noble metal-comparable SERS enhancement from semiconducting metal oxides by making oxygen vacancies. *Nat. Commun.* **2015**, 6, 7800.

2. Tao, L.; Chen, K.; Chen, Z.; Cong, C.; Qiu, C.; Chen, J.; Wang, X.; Chen, H.; Yu, T.; Xie, W.; Deng, S.; Xu, J. B. 1T′ transition metal telluride atomic layers for plasmon-free SERS at femtomolar levels. *J. Am. Chem. Soc.* **2018,** 140, 8696–8704.

3. Liang, C.; Lu, Z. A.; Zheng, M.; Cheng, M. X.; Zhang, Y. Y.; Zhang, B.; Zhang, J. X.; Xu, P. Band structure engineering within two-dimensional borocarbonitride nanosheets for surface-

enhanced Raman scattering. *Nano Lett.* **2022**, 22, 6590–6598.

4. Tang, X.; Hao, Q.; Hou, X.; Lan, L.; Li, M.; Yao, L.; Zhao, X.; Ni, Z.; Fan, X.; Qiu, T. Exploring and Engineering 2D Transition Metal Dichalcogenides toward Ultimate SERS Performance. *Adv. Mater.* **2024**, 36, 2312348.
5. Zhao, S.; Wang, H.; Niu, L.; Xiong, W.; Chen, Y.; Zeng, M.; Yuan, S.; Fu, L. 2D GaN for highly reproducible surface enhanced Raman scattering. *Small* **2021**, 17, 2103442.
6. Li, Y.; Chen, H.; Guo, Y.; Wang, K.; Zhang, Y.; Lan, P.; Guo, J.; Zhang, W.; Zhong, H.; Guo, Z.; Zhuang, Z. Lamellar hafnium ditelluride as an ultrasensitive surface-enhanced Raman scattering platform for label-free detection of uric acid. *Photonics Res.* **2021**, 9, 1039–1047.
7. Shao, M.; Ji, C.; Tan, J.; Du, B.; Zhao, X.; Yu, J.; Man, B.; Xu, K.; Zhang, C.; Li, Z. Ferroelectrically modulate the Fermi level of graphene oxide to enhance SERS response. *Opto-Electron. Adv.* **2023**, 6, 230094.
8. Lan, L.; Hou, X.; Gao, Y.; Fan, X.; Qiu, T. Inkjet-printed paper-based semiconducting substrates for surface-enhanced Raman spectroscopy. *Nanotechnology* **2020**, 31, 055502.
9. Lan, L.; Yang, S.; Li, G.; Zhao, C.; Liu, J.; Zhao, X.; Qu, Z.; Gao, J.; Fan, X.; Qiu, T. Direct writing of flexible two-dimensional MXene arrays for SERS sensing. *J. Mater. Chem. C* **2024**, 12, 12115–12123.
10. Ge, Y.; Wang, F.; Yang, Y.; Xu, Y.; Ye, Y.; Cai, Y.; Zhang, Q.; Cai, S.; Jiang, D.; Liu, X.; Liedberg, B. Atomically thin $TaSe_2$ film as a high-performance substrate for surface-enhanced Raman scattering. *Small* **2022**, 18, 2107027.
11. Wang, L.; Yu, D.; Huang, B.; Ou, Z.; Tao, L.; Tao, L.; Zheng, Z.; Liu, J.; Yang, Y.; Wei, A.; Zhao, Y. Large-area ReS_2 monolayer films on flexible substrate for SERS based molecular sensing with strong fluorescence quenching. *Appl. Surf. Sci.* **2021**, 542, 148757.
12. Lan, L.; Ni, Z.; Zhao, C.; Gao, J.; Tang, X.; Qu, Z; Zheng, L., Fan, X.; Qiu, Teng. Photoinduced charge transfer empowers Ta_4C_3 and Nb_4C_3 MXenes with high SERS performance. *Langmuir* **2024**, 40, 20945–20953.
13. Meng, L.; Hu, S.; Xu, C.; Wang, X.; Li, H.; Yan, X. Surface enhanced Raman effect on CVD growth of WS2 film. *Chem. Phys. Lett.* **2018**, 707, 71–74.
14. Guan, Y.; Chen, M.; Ding, Y.; Fang, Y.; Huang, F.; Xu, C.Y.; Zhen, L.; Li, Y.; Yang, L.; Xu,

P. Phase transformation on multilayer 2M-WS_2 for improved surface-enhanced Raman scattering. *ACS Nano* **2024**, 18, 17339–17348.
